# Unveiling environmental transmission risks: comparative analysis of azole resistance in *Aspergillus fumigatus* clinical and environmental isolates from Yunnan, China

**DOI:** 10.1128/spectrum.01594-24

**Published:** 2024-10-29

**Authors:** Jianchuan Gong, Jiarui Huang, Yongju Liu, Ying Zhang, Yuhong Gao

**Affiliations:** 1 State Key Laboratory for Conservation and Utilization of Bio-Resources in Yunnan, Yunnan University, Chenggong District, Kunming, Yunnan, China; 2 College of Life Science, Yunnan University, Chenggong District, Kunming, Yunnan, China; 3 Department of clinical laboratory, The First People’s Hospital of Yunnan Province, Xishan District, Kunming, Yunnan, China; Universidade de Sao Paulo, Ribeirao Preto, Sao Paulo, Brazil

**Keywords:** antifungal resistance, endemic mycoses, environmental transmission

## Abstract

Azole resistance in *Aspergillus fumigatus* poses a significant clinical challenge globally. Our previous epidemiological analysis revealed a remarkably high frequency (~80%) of azole-resistant *A. fumigatus* in Yunnan’s greenhouse environments, prompting increased local and regional research for targeted control strategies. In this study, we analyzed 94 clinical *A. fumigatus* isolates from Yunnan, comparing their susceptibility profiles and genotypic characteristics with environmental strains previously isolated. While the overall frequency of azole resistance in clinical isolates was lower than that in environmental samples, a significant prevalence of cross-resistance, with varying resistance patterns based on minimum inhibitory concentration (MIC) levels was observed, which exceeded rates in other regions of China. Specific mutation combinations in the *cyp51A* gene were linked to elevated MIC values in clinical and/or environmental samples, while some resistant strains with wild-type *cyp51A* remain unexplained, indicating a need for further investigation into their resistance mechanisms. The differences in unique genetic elements and the distinct genetic differentiation observed between clinical and environmental isolates can be attributed to Yunnan’s unique geomorphology and potential genotype importation from other provinces and abroad. Extensive allele exchanges and sharing contributed to the selection of azole-resistant clinical isolates, suggesting a common environmental origin, and the transmission routes of local drug-resistant strains cannot be excluded. These findings emphasize the imperative for regional and targeted surveillance to monitor resistance trends and guide effective antifungal therapy, and management strategies to mitigate invasive aspergillosis risk in this region.

**IMPORTANCE:**

Azole resistance in *Aspergillus fumigatus* is a major global health concern, with particularly high rates (~80%) observed in Yunnan’s greenhouse environments. This study compares azole resistance in 94 clinical isolates from Yunnan with environmental strains, revealing lower clinical resistance but significant cross-resistance and distinct resistance patterns. Specific mutations in the *cyp51A* gene were associated with elevated minimum inhibitory concentration values, though some resistant strains had wild-type *cyp51A*, highlighting the need for further research. The unique genetic profiles and potential external genotype influences in Yunnan emphasize the need for targeted regional surveillance. Effective monitoring and control strategies are essential to manage and mitigate the risk of invasive aspergillosis.

## INTRODUCTION

As a saprophytic fungus characterized by airborne conidia with a diameter of 2.5–3.5 μm and a rapid reproductive rate, *Aspergillus fumigatus* is widely distributed across the globe, inhabiting diverse environments such as soil, air, water, and plant rhizomes (1–3). *A. fumigatus* ranks among the most prevalent fungal pathogens, causing a spectrum of aspergillosis infections and emerging as the second most common invasive mycosis in hospital settings, trailing only *Candida* (3, 4). Particularly concerning is its potential to induce fatal invasive aspergillosis (IA) in immunocompromised individuals (5). Despite being pivotal in clinical management, first-line drugs such as itraconazole (ITR) and voriconazole (VOR) face challenges due to the increasing emergence of azole-resistant *A. fumigatus* (AR*Af*) strains. This trend has substantially elevated treatment failure rates and mortality rates, reaching up to 95% in immunocompromised hosts, thereby posing a grave threat to both human health and environmental well-being (6, 7).

Two primary pathways contribute to the development of resistance in *A. fumigatus*, both stemming from the prolonged and widespread use of antifungal medications (8). First, there is the patient-acquired pathway, wherein drug-resistant strains emerge from individuals subjected to long-term azole treatments. The second pathway, the environmentally acquired one, arises independently. However, the potential for cross-transmission between these two pathways remains plausible (9, 10). *A. fumigatus* primarily spreads through spores, facilitated by human social interactions, enabling its dissemination across vast distances (7). Van der Linden et al. identified azole-resistant *A. fumigatus* strains among patients lacking prior clinical azole exposure in the Netherlands during 2007–2009, suggesting environmental-to-clinical transmission (11). Similarly, studies in Iran confirmed the spread of environmentally azole-resistant *A. fumigatus*, sharing identical alleles with clinical isolates (2). France also reported identical azole-resistant genotypes in *A. fumigatus* from various sources, including vegetable gardens, sawmills, and clinical patients (12). Moreover, Lofgren et al. demonstrated that while asexual spores facilitate the transmission of *A. fumigatus* strains, extensive genetic recombination occurs among them (13). Furthermore, the entry of *A. fumigatus* spores from patients’ lungs into the environment represents another potential transmission route from clinical settings (14). The same genotype detected in cough plates and sputum samples suggests that *A. fumigatus* may be aerosolized in humans and that droplet or airborne transmission may be a potential route of transmission between patients (15). In China, epidemiological analyses by Chen et al. revealed matching mutation mechanisms and genotypes between *A. fumigatus* isolated from hospital soil samples and clinical sources in Shenyang and Chengdu (16). Similarly, Xu et al. isolated *A. fumigatus* from clinical patients with possible environmental origins of azole resistance (17). Cao et al.’s investigations in eastern China from 2014 to 2018 revealed a prevalence of non-wild-type *A. fumigatus* exceeding 20% in hospital settings, signifying a high risk of susceptible patients inhaling spores and developing azole-resistant aspergillosis (18).

Resistance mechanisms in *A. fumigatus* are diverse, with the most prevalent involving mutations in the *cyp51A* gene. This gene, crucial for ergosterol biosynthesis, encodes lanosterol 14-α-demethylase, a key target for azoles (7, 19). Mutations in *cyp51A* can lead to amino acid substitutions, resulting in various degrees of triazole resistance, influenced by mutation site and tandem repeat insertion in the upstream regulatory region. Notably, mutations at positions G54, L98, Y121, G138, P216, F219, M220, A284, and T289 are frequently associated with triazole resistance, with non-synonymous mutations at positions 98, 121, and 289 often accompanied by tandem repeats in the promoter region (20–22). The TR_34_/L98H mutation stands out as the most common resistance mutation in both environmental and clinical *A. fumigatus* strains. First identified in the Netherlands in 1998, it has since been reported in several countries, including France, the United Kingdom, Germany, China, and Switzerland (23–27). Similarly, the TR_46_/Y121F/T289A mutation, discovered in the Netherlands in 2012, has been detected in Belgium, Tanzania, India, Germany, Japan, Denmark, Spain, and Switzerland (27–33).

In addition to TR_34_/L98H and TR46/Y121F/T289A, mutations such as G448S, TR_53_, TR_34_/R65K/L98H, and F46Y/M172V/E427K have been linked to AR*Af* (6) (34). In China, TR_34_/L98H/V242I/S297T/F495I and TR_46_/Y121F/V242I/T289A have been observed (26). Notably, studies in eastern China revealed TR_34_/L98H and TR_46_/Y121F/T289A among clinical isolates, while a tertiary hospital in Ningxia found the L98H/S297T mutation (35, 36). In Yunnan, southwest China, high-frequency environmental AR*Af* strains displayed TR_34_/L98H, TR_46_/Y121F/T289A, and TR_53_ mutations (37). In one study, three out of five patients with invasive pulmonary aspergillosis in Yunnan carried the TR_34_/L98H mutation, one had TR_46_/Y121F/T289A, and another TR_34_/L98H/S297T/F495I (17). These findings suggest TR_34_/L98H and TR_46_/Y121F/T289A as predominant mutations in AR*Af* in Yunnan, though the presence of other mutation types indicates a complex landscape. While environmental TR_53_ mutation was detected for the first time in Asia, its spread to clinical samples in Yunnan remains uncertain. Large-scale investigations are needed to elucidate AR*Af* transmission and resistance mechanisms in Yunnan’s clinical and environmental settings.

In recent years, the population genetic structure of *A. fumigatus* across diverse geographic regions and ecological niches has been extensively investigated, employing various molecular markers to elucidate the origins, differentiation, and dissemination of drug-resistant strains (38–40). Microsatellite genotyping has proven instrumental in identifying potential reservoirs of resistance genotypes for *A. fumigatus* and understanding how these resistant genotypes are shared among patients, geographic regions, countries, and continents (41). Sewell et al. analyzed 4,049 *A*. *fumigatus* isolates from global sources using 9 highly polymorphic microsatellite markers, revealing 2 distinct genetic populations (42). Similarly, a binary genetic differentiation pattern was observed in a detailed indoor sampling study of *A. fumigatus* at Walailak University, suggesting the potential spread of *A. fumigatus* from a single ancestor to multiple sites within the study area (43). Barber et al. conducted a genetic diversity analysis of 83 clinical and 217 environmental isolates, revealing 3 genetic populations among clinical and environmental *A. fumigatus* isolates, with a significant overlap between clinical and environmental strains (44). In Yunnan Province, two distinct genetic populations were identified through fine-scale and large-scale sampling in greenhouses and remote areas, respectively. These findings were corroborated by combined analyses with strains isolated globally, suggesting an independent genetic differentiation clade from Yunnan (37, 40, 45).

Yunnan Province, a prominent agricultural region in China, boasts vast production of vegetables, fruits, and flowers, catering to domestic and international markets. Renowned as a global biodiversity hotspot (46), Yunnan faces a unique challenge regarding *A. fumigatus* resistance, particularly within its greenhouse environments. Here, we observed an unprecedented AR*Af* frequency of approximately 80%, marking the highest recorded occurrence globally (37). In contrast, within the province’s diverse ecological niches—including farmlands, orchards, lakes, and forests—AR*Af* prevalence stands at 15.9% (45). While significantly lower than the greenhouse population, this figure still surpasses resistance frequencies observed in other Chinese regions. Notably, these *A. fumigatus* populations exhibit substantial genetic diversity, marked genetic differentiations, and independent evolutionary trajectories compared to global counterparts. The abundance of novel genotypes necessitates the exploration of their origins and transmission routes.

Our study aims to first assess *A. fumigatus* resistance and its mechanisms within clinical samples from the same areas. Subsequently, we seek to unravel the transmission pathways of frequently encountered AR*Af* isolates in Yunnan Province and delve into the genetic interplay between greenhouse, clinical, and environmental samples. These endeavors will shed light on the necessity of establishing triazole resistance surveillance, providing robust evidence to comprehend the acquisition and dissemination of environmental *A. fumigatus* triazole resistance. Ultimately, such insights will uphold the principles of “One Health,” ensuring the well-being of both human and environmental ecosystems.

## MATERIALS AND METHODS

### Source, isolation, and identification of strains

Sputum and bronchoalveolar lavage fluid (BALF) from patients with pulmonary infection were collected at the First People’s Hospital of Yunnan Province from 2020 to 2023, where most patients with IA in Yunnan Province were treated.

Specificially, specimens, including morning sputum, BALF obtained via bronchoscopy, and induced sputum (5 mL), were transported to the laboratory within 2 hours in sterile sputum cups. Sputum quality was assessed by microscopic evaluation, requiring <10 squamous epithelial cells and >25 white blood cells per low-power field, which helped to ensure that the sputum specimen was not saliva or nasopharyngeal secretions but truly from the lower respiratory tract. BALF specimens were deemed acceptable if they contained <1% squamous epithelial cells, <5% columnar epithelial cells, and <10% red blood cells. In a biological safety cabinet, sputum and BALF samples were inoculated onto blood agar, Sabouraud agar, chocolate agar, and MacConkey agar, streaked in three zones. Plates were incubated at 35°C for 48–72 hours for Sabouraud and MacConkey agars, and in a CO_2_ incubator for 48 hours for blood and chocolate agars. After incubation, colonies appeared as fluffy or cottony, with colors ranging from smoky green or blue-green to white or yellowish-white and from at least two of the four agar plates where more than two colonies were present in the original streak zone, were transferred to five points onto potato dextrose agar and Sabouraud dextrose agar plates for further purification and identification.

The microscopic and macroscopic morphological characteristics of the isolated *A. fumigatus* were observed. For more accurate identification, we extracted the genomic DNA from mature hyphae of the putative *A. fumigatus* cultures using the modified cetyltrimethylammonium bromide method (47). The extracted genomic DNAs were used as templates to amplify and sequence the internal transcribed spacer (ITS) regions with primer pairs ITS4 and ITS5 (48). The obtained sequences were compared with known sequences of *A. fumigatus* on central BLAST repositories in NCBI to ensure their species identification (49, 50). The accuracy of identification was further confirmed by matrix-assisted laser desorption ionization-time of flight (MALDI-TOF) mass spectrometry, which linked to a reference database (Vitek MS Knowledge Base, v3.0) (51). Sample preparation was performed according to the manufacturer’s instructions using the Vitek MS Mold Reagent Kit (bioMérieux, Durham, NC). The environmental samples were taken from the study of Zhou et al. (45), in which 251 isolates of *A. fumigatus* were selected and consistent with the source of the clinical patient’s residence.

### Susceptibility of *A. fumigatus* isolates

We employed CLSI M38-A2 and M27-A4 methods to assess the sensitivity of each strain to selected drugs. Initially, identified *A. fumigatus* strains were activated, and their spores were harvested in a saline solution containing Tween 20, utilizing disposable swabs. These spores were then prepared into a 0.5-Mcf (millions of colony-forming units; 5 × 10^6^ conidia/mL) suspension with the assistance of a turbidimeter (DensiCHEK Plus from msamrieux, USA). Mcf is a unit used to express the concentration of microbial cells in a solution. For example, a concentration of 5 Mcf/mL means there are 5 million colony-forming units per milliliter of the solution. For *in vitro* susceptibility testing, we utilized a fungal susceptibility test kit manufactured by Autobio Diagnostics, capable of simultaneously assessing the sensitivity of 10 drugs. The test followed the microbroth dilution method, where reference strains *Candida parapsilosis* (ATCC22019) and *C. krusei* (AT6258) served as controls. Each drug-sensitive plate provided a corresponding fungal culture and color development solution, with individual wells representing different drug concentrations. Minimum inhibitory concentration (MIC) readings were determined based on color and turbidity changes of the medium post-incubation.

For ITR, posaconazole (POS), VOR, and isavuconazole, the MIC is determined as the lowest concentration of the drug that completely inhibits any observable growth (100% inhibition). On the other hand, for echinocandins [anidulafungin (ANI), caspofungin (CAS), micafungin], the minimum effective concentration (MEC) is identified as the lowest concentration at which colonies on the surface of the microwells exhibit non-confluent growth, contrasting with the turbid growth observed in the control wells. In cases where the endpoint of MEC growth is unclear, it can be confirmed as MEC by observing the lowest concentration at which hyphae exhibit compact and rounded growth under microscopic examination. To prevent drug or spore suspension volatilization, approximately 30 µL of mineral oil was added post-sample addition. MIC/MEC determination for *A. fumigatus* isolates with each drug was conducted at least three times on different days, encompassing all reference strains.

### Sequencing and analyses of *cyp51* gene and its upstream sequence

Primers cyp51A-F(5′-AGTTCCAGCATACCATACAC-3′) and cyp51A-R (5′-CCTATTCCGATCACACCAA −3′) were used to amplify the cyp51 amino acid-coding gene of *A. fumigatus*. Primers P-A5-F (5′-TCTCTGCACGCAAAGAAGAAC-3′) and P-A7-R (5′-TCATATGTTGCTCAGCGG-3′) were used to amplify the upstream sequence of *cyp51A*. Mutations of *cyp51A* gene were identified by comparing our sequences with the reference sequence of a triazole-susceptible *A. fumigatus* strain in GenBank with the accession number AF338659. The mating type of each strain was identified through PCR using primers AFM1 (5′-CCTTGACGCGATGGGGTGG-3′), AFM2 (5′-CGCTCCTCATCAGAACAACTCG-3′), and AFM3 (5′-CGGAAATCTGATGTCGCCACG-3′) (52).

### Statistical analyses of population genotype data between clinical and environmental samples

We used the panel of nine highly polymorphic short tandem repeat (STR) markers as previously described to genotype all newly isolated clinical *A. fumigatus* strains (53). The number of tandem repeats at each locus for each strain was determined based on fragment length comparison, following previously described protocols (37). The combined allelic information at the nine STR loci constitutes the multilocus genotype (MLG) of each strain. The STR data set including environmental *A. fumigatus* isolates from our previous study (45).

To assess the proportion of genetic variation within and between clinical and environmental populations in Yunnan Province, molecular analysis of variance (AMOVA) was performed using GenAlEx 6.5 (54). The null hypothesis of AMOVA is that there are no genetic differences between test populations. For the calculation of genetic distances between strains, the parameter haploid-STR was used to merge stepwise mutation models. Genetic distance is represented by the differences in the repeats number of alleles at each locus. Genetic differentiation between the pairwise populations was calculated using Fst determined by 999 permutations (52). In addition, GenAlEx 6.5 was also used to evaluate the population genetic diversity using Shannon’s index (*I*) and Shannon’s mutual information index not only offers an easy way to measure differences between populations, but it can also be converted into the log-likelihood contingency test G statistic. This conversion allows for a straightforward chi-square test (χ² test) to compare allele frequencies between pairs of populations (54). The program STRUCTURE version 2.3.5 (55) was used to determine the optimal number (*K*) of genetic clusters in all samples of Yunnan Province.

To visualize the relationships between MLGs from clinical and environmental *A. fumigatus* isolates, a minimum spanning network (MSN) was generated by calculating Bruvo’s genetic distance between strains using the R package poppr (56). Bruvo’s genetic distance is specific for STR genotypes and incorporates the stepwise mutation model. In addition, a multivariate analysis, discriminant analysis of principal components (DAPC), and a principal coordinate analysis (PCoA) implemented by adegenet package in R were used to cluster MLG genotypes in relation to their geographic origins (57).

### Statistical analysis

The chi-square test was used to evaluate the correlation association between the observed *cyp51A* gene mutations and ranges of MICs, as well as the geographical origins of isolates. *P* < 0.05 was considered statistically significant. It was performed using GraphPad Prism software version 5 (GraphPad Software Inc., San Diego, CA, USA).

## RESULTS

### Isolation and susceptibility of *A. fumigatus* isolates

A comprehensive collection of 6,700 specimens, including sputum, BALF, and tracheal and bronchial inhalations, was amassed from patients diagnosed with lung infections at the First People’s Hospital of Yunnan Province. This hospital serves as a primary facility for lung disease patients across Yunnan Province. A total of 94 clinical strains of *A. fumigatus* were subsequently isolated and identified through a combination of morphological characterization, internal transcribed spacer sequencing, and MALDI-TOF mass spectrometry; all our clinical strains showed 99.9% similarity to the reference strain of *A. fumigatus* in the Vitek MS Knowledge Base, v3.0. Successful genotyping using nine *A. fumigatus*-specific primers was also achieved, which further confirmed the identification of this clinical strains. Additionally, 251 environmental isolates of *A. fumigatus* were randomly selected from 19 populations as described by Zhou et al. (45). These isolates, along with the clinical strains, were categorized into eight distinct populations based on their sources and geographic regions. These populations include clinical samples from central Yunnan (CC), environmental samples from central Yunnan (CE), clinical samples from eastern Yunnan (EC), environmental samples from eastern Yunnan (EE), clinical samples from western Yunnan (WC), environmental samples from western Yunnan (WE), clinical samples from southern Yunnan (SC), and environmental samples from southern Yunnan (SE) (see Table 1).

**TABLE 1:** Prevalence of azole resistance for 345 clinical and environmental *A. fumigatus* isolates from in Yunnan

Population	No. of isolates	ITR (≥2)^*a*^	VOR (≥2)	ITR (≥4)	VOR (≥4)	ANI (≥0.03)	CAS (≥1)	MIF^d^ (≥0.06)	AMB^*d*^ (≥4)	POS (≥0.25)	NYS^*d*^ (≥8)
CC	61	8 (13.11) ^*b*^	8 (13.11)	4 (6.56)	4 (6.56)	3 (4.92)	1 (1.64)	1 (1.64)	5 (8.2)	44 (63.93)	60 (98.36)
CE	43	7 (16.28)	12 (27.91)	6 (13.95)	9 (20.93)	n/a^e^	n/a^e^	n/a^e^	n/a^e^	n/a^e^	n/a^e^
WC	7	0	0	0	0	4 (57.14)	0	0	0	4 (57.14)	7 (100)
WE	61	15 (24.59)	16 (26.23)	11 (18.03)	11 (18.03)	n/a^e^	n/a^e^	n/a^e^	n/a^e^	n/a^e^	n/a^e^
EC	17	5 (29.41)	8 (47.06)	4 (23.53)	6 (35.29)	6 (35.29)	0	1 (5.88)	1 (5.88)	11 (64.71)	17 (100)
EE	40	8 (20)	6 (15)	7 (17.5)	3 (7.5)	n/a^e^	n/a^e^	n/a^e^	n/a^e^	n/a^e^	n/a^e^
SC	9	2 (22.22)	4 (44.44)	0	3 (33.33)	4 (44.44)	0	0	1 (11.11)	8 (88.88)	9 (100)
SE	107	17 (15.89)	11 (10.28)	12 (11.21)	4 (3.74)	n/a^e^	n/a^e^	n/a^e^	n/a^e^	n/a^e^	n/a^e^
Total	345	61 (17.68)	65 (18.84)	44 (11.88)	40 (11.59)	17 (10.09) ^*c*^	1 (1.06)	2 (2.13)	7 (7.45)	67 (71.28)	93 (98.94)

^*a*^ MIC value: mg/L.

^*b*^ No. of resistant isolates, the percentage of them in the population is in brackets, and the percentile sign (%) has been omitted.

^*c*^ Since there are no data on the resistance of environmental strains to ANI, CAS, MIF, AMB, POS, and NYS, the denominator of the resistance frequency of clinical strains to these drugs is the total number of clinical strains—94.

^*d*^ NYS, nystatin; AMB, amphotericin; MIF, micafungin

^*e*^ Not applicable.

The results of *in vitro* drug susceptibility testing for all clinical *A. fumigatus* strains revealed intrinsic resistance to fluconazole and fluorocytosine across the sampling areas, with only one clinical strain from central area resistant to caspofungin. However, another clinical strain from the same area displaying susceptibility to nystatin tablets (NYS), which is the only susceptible strain to NYS in our study. In the four geographical regions, the clinical strains from the eastern area had higher susceptibility values of 2 and 4 mg/L to both ITR and VOR than the environmental strains, while the opposite was true in the other regions, that is, the frequency of resistance to ITR and VOR was higher in the environmental strains than in the clinical strains (Table 1). Although no individuals with MIC values greater than 2 mg/L were found in the clinical strains from the western area, the distribution frequency of strains with ITR of ≥2 mg/L (24.59%) and 4 mg/L (18.03%) was found to be highest in the same area with environmental origin. Resistance frequencies to different drugs varied among clinical samples, with the highest resistance observed to POS at 71.28% except for the aforementioned NYS, followed by VOR at 14.89% (14/94) and ITR at 9.57% (9/94). Remarkably, resistance prevalence to ITR and VOR varied significantly across different regions and origins. Notably, the highest resistance to ITR at both 2 and 4 mg/L was found in the EC population (29.41% and 23.52%), followed by WE (24.59% and 18.03%), while all *A. fumigatus* strains in WC remained susceptible to both ITR and VOR. The highest prevalence of VOR resistance at both 2 and 4 mg/L was also observed in EC (47.46% and 35.29%), followed by SC (44.44% and 33.33%). Meanwhile, the EC population showed the highest frequency of resistant to Micafungin (MIF) (0.06 mg/L, 5.88%), with the lowest frequency of resistance observed to amphotericin (AMB) at 4 mg/L (5.88%). Additionally, the southern region of clinical population displayed the highest frequency of clinical *A. fumigatus* isolates resistant to POS (88.89%).

In addition, our study highlights the prevalence and variability of cross-resistance to itraconazole and voriconazole in *A. fumigatus* across different MIC thresholds. At MICs ≥2 mg/L, cross-resistance to both ITR and VOR was observed in 13.83% of the strains. This rate decreased to 4.26% when MICs reached or exceeded 4 mg/L. Notably, 2.13% showed cross-resistance to amphotericin when the MICs of ITR and VOR are ≥2 mg/L, and 1.06% of the strains exhibited cross-resistance to AMB and anidulafungin, while 12.77% displayed resistance to POS and AMB. At higher MICs (≥4 mg/L), cross-resistance to AMB and ITR was observed in 1.06%.

### The occurrence of *cyp51A* gene mutations and their geographic distributions

In our previous study on environmental *A. fumigatus* in Yunnan, we only amplified the promoter region and coding region of the *cyp51A* gene in resistant strains. To comprehensively elucidate the variation and distribution of resistance target genes in environmental and clinical strains in Yunnan, this study successfully amplified all the aforementioned sequences for all 94 clinical and the selected 251 environmental strains. The occurrence of tandem repeat insertion in the promoter region of the *cyp51A* gene was 6.09% (21/345), including TR_34_ (18/345, 5.22%) and TR_46_ (3/345, 0.87%). While TR_46_/Y121F and TR_34_/L98H/S297T/F495I tandem repeats and mutation combinations were found only in clinical samples, more TR_34_/L98H combinations were found in environmental samples than clinical ones (12 vs 6), and it distributed across different geographic areas except for the clinical samples from west and south. The abovementioned types of mutations occurred in 27.27% of the CC population, while only 4.55% occurred in the SE and EE populations. No such tandem repeat insertions were found in WC and SC (see Table 2).

**TABLE 2:** *cyp51A* gene amino acid and the promoter information from all the 345 *A*. *fumigatus* isolates from different population

Pop	No. of isolates	Mutation type								
		TR34/	TR34/L98H/	TR46/	F46Y	M172V	N248T/K	D255E	E427K	Tandem repeat insertion
		L98H	S297T/F495I	Y121F						
CC	61	4 (6.56%)	1 (1.64%)	1 (1.64%)			7 (11.48%)		1 (1.64%)	6 (9.84%)
CE	43	5 (11.63%)					3 (6.98%)			5 (11.63%)
WC	7						1 (14.29%)			0
WE	61	5 (8.20%)			4 (6.56%)	4 (6.56%)	12 (19.67%)	3 (4.92%)	4 (6.56%)	5 (8.20%)
EC	17	2 (11.76%)					2 (11.76%)		1 (5.88%)	2 (11.76%)
EE	40	1 (2.50%)			1 (2.50%)	1 (2.50%)	4 (10.00%)	1 (2.50%)	1 (2.50%)	1 (2.50%)
SC	9			2 (22.22%)			3 (33.33%)			2 (22.22%)
SE	107	1 (0.93%)			4 (3.74%)	5 (4.67%)	18 (16.82%)	3 (2.80%)	4 (3.74%)	1 (0.93%)
Total	345	18 (5.22%)	1 (0.29%)	3 (0.87%)	9 (2.61%)	10 (2.90%)	50 (14.49%)	7 (2.03%)	11 (3.19%)	22 (6.38%)

There are nine non-synonymous substitutions (F46Y, L98H, Y121F, M172V, N248T/K, D255E, S297T, E427K, and F495I) in our data set with 345 isolates. Among them, Y121F, S297T, and F495I were exclusively found in clinical samples, while F46Y, M172V, and D255E only occurred in environmental samples. A combination of five non-synonymous substitutions—F46Y, M172V, N248T, D255E, and E427K—was present in 7 of the 345 strains (2.03%), 2 of them were cross-resistant to ITR and VOR with MIC higher than 2 mg/L, and 2 were either resistant to ITR or VOR, and the remaining 3 strains were susceptible. Additionally, the F46Y, M172V, and K427E combination had an incidence of 0.58% (2/345). The F46Y/M172V/N248T/D255E/E427K and F46Y/M172V/E427K combinations were exclusively found in environment samples.

In particular, 46 *A*. *fumigatus* isolates had singleton mutations. Among them, 38 clinical and environmental strains had N248K mutation, 5 strains from WE had N248E mutation, 2 clinical strains had E427K mutation, and 1 strain had M172V mutation.

There were 268 WT *cyp51A* strains in total 345 samples. Among them, 32 and 21 were found to have MIC value ≥2 and 4 mg/L for ITR, 35 and 18 were found to have MIC value ≥2 and 4 mg/L for VOR, both from clinical and environmental origins. Furthermore, with 17 and 7 isolates have MIC value ≥2 and 4 mg/L for both ITR and VOR, among the latter 7 isolates with high MIC values, 1 clinical strain also had high MIC against AMB (8 mg/L) and POS (1 mg/L), respectively.

The chi-square test was used to evaluate the correlation between the observation of *cyp51A* gene mutations, their geographic origins, and MIC ranges. The results showed that the TR_34_ insertion was more likely to occur in central Yunnan and western Yunnan (*P* = 0.006, *P* = 0.000), while TR_46_ was more likely to occur in central Yunnan (*P* = 0.008). Additionally, we found that the occurrence of the TR_34_ mutation type did not significantly differ between clinical and environmental samples (*P* = 0.334). However, the TR_46_ mutation was only found in clinical samples, while the occurrence of other mutation types did not significantly differ between clinical and environmental samples.

TR_34_/L98H mutation shows a significant correlation with both clinical and environmental strains regarding resistance to ITR and VOR, though the range of MIC values for these associations differs. For instance, in clinical strains, the association with ITR resistance is present when MIC is greater than 1 mg/L (*P* = 0), whereas the association with VOR resistance is broader, starting from 0.125 mg/L (*P* = 0). In contrast, for environmental strains, the association pattern is reversed: the correlation with ITR resistance is broader, present at MIC values greater than 0.5 mg/L (*P* = 0), while for VOR resistance, a significant association is only observed at MIC values greater than 1 (*P* = 0.01). The TR_46_/Y121F mutation is present only in clinical strains and shows a significant correlation exclusively with high MIC values (greater than 8 mg/L) against VOR (*P* = 0.003).

The mutations TR_34_/L98H/S297T/F495I, which occur only in clinical strains, are significantly associated with ITR MIC values greater than 1 mg/L (*P* = 0.016) and VOR MIC values greater than 4 mg/L (*P* = 0.012). For mutations found exclusively in environmental strains, the F46Y/M172V/E427K combination shows a significant association only with ITR MIC values greater than 16 mg/L (*P* = 0.038), while the F46Y/M172V/N248T/D255E/E427K combination is significantly associated with ITR MIC values greater than 0.5 mg/L (*P* = 0.01) and VOR MIC values greater than 1 mg/L (*P* = 0.006). N248K is widely present in both clinical and environmental strains without a significant association with any specific MIC value, whereas N248T, present only in environmental strains, is significantly associated with lower MIC values for ITR and VOR, such as 0.25 mg/L (*P* = 0.002) and 0.5 mg/L (*P* = 0.023). The occurrence of mutation types is also related to the resistance of clinical isolates to several clinical drugs. For example, the TR_34_/L98H mutation is significantly associated with MIC values of other clinical drugs at low concentrations, such as ANI (MIC = 0.008 mg/L, *P* = 0) and POS (MIC = 0.25 and 0.5 mg/L, *P* = 0). The mutation combination TR_34_/L98H/S297T/F495I is closely associated with POS (MIC = 1 mg/L, *P* = 0), while TR_46_/Y121F is closely linked to POS at MIC values of 0.25 mg/L (*P* = 0.01) and 1 mg/L (*P* = 0.01). Although the N248K mutation is widespread in both clinical and environmental strains, it is associated with resistance to ANI (0.03 mg/L, *P* = 0.04), MIF (0.008 mg/L, *P* = 0), and AMB (0.5 and 1 mg/L, *P* = 0.001) specifically in clinical strains.

### Genotyping of *A. fumigatus* isolates and population genetic analyses

There were differences in genetic diversity between environmental and clinical samples; the Shannon index between clinical and environmental samples was found to be significantly different by *t*-test (*P* < 0.001). The SE population had the highest Shannon index (I) of 2.459, while the WC population had the lowest at 1.573. The unbiased diversity (uh) ranged between 0.844 and 0.893 (Table S1). Among the 237 alleles identified from the 345 strains isolated from 8 populations, the SE population had the highest number of private alleles (10) followed by the CC population (8). No private alleles were found in the SC population.

Among the nine STR loci, the number of alleles in clinical *A. fumigatus* isolates was smaller than that in environmental samples. All alleles at locus 2A were shared between clinical and environmental samples, whereas the highest number of private alleles was found at loci 3A (7) and 3C (7) (Table 3). We also identified 317 MLGs. Only 22 MLGs were shared among at least 2 populations, with no clinical and environmental samples sharing identical MLGs. However, MLGs were shared within different geographic regions and origins, with the highest frequency in SE (14), followed by CE (9), and the lowest in SC (2). In addition, there were 14 private alleles and 220 unique MLGs identified in environmental populations, accounting for 6.39% (14/219) of the total environmental alleles and 94.02% (220/234) of the total environmental MLGs, respectively. While the clinical groups exhibited lower frequency of private genetic elements, with 5.92% private alleles (10/169) and 88.37% unique MLGs (76/86).

**TABLE 3:** STR allele distributions and genetic diversity within and among the eight populations of *A. fumigatus* for each of the nine STR loci

Loci	No. of alleles	No. of population alleles (no. of private alleles)							
		CE	CC	WE	WC	EE	EC	SE	SC
SSRAF2A	17	13	11	12	7	12	10	16	5
SSRAF2B	25	15	14 (1)	17 (2)	4	13	9	18 (1)	5
SSRAF2C	28	16 (1)	18 (2)	16	7 (1)	15 (1)	10 (1)	20	7
SSRAF3A	49	27 (2)	23	29	6	24	11 (1)	39 (4)	7
SSRAF3B	32	16	21 (2)	21 (2)	7	19	12 (1)	25	7
SSRAF3C	38	20	27 (2)	18	7	19	13 (1)	33 (4)	7
SSRAF4A	20	12 (1)	14	14 (2)	5	9 (1)	7	13	7
SSRAF4B	13	9 (1)	9 (1)	9 (1)	3	6	7	10 (1)	3
SSRAF4C	15	7	8	9	3	8 (2)	6 (1)	8	4
Total	237	135 (5)	145 (8)	145 (7)	49 (1)	125 (4)	85 (5)	182 (10)	52

Notably, we found several pairs of clinical and environmental strains with similar alleles: 2205NY0098 and GN5, 2211SP0711 and LL5 each pairs have six identical alleles, strains from the latter pair are both ITR and VOR resistant, and the environmental strains (GN5 and LL5) are from southern and eastern Yunnan, respectively; 2304SP0782 and JH17, 2205SP0567 and LC22, and 2212SP0400 and GN23 each pairs have five identical alleles, all environmental strains (JH17, LC22, and GN23) are from southern Yunnan too. Other environmental strains with two to four alleles sharing with clinical strains occurred at Luxi, Lincang, and Guangnan counties from southern Yunnan, Luliang counties from eastern Yunnan, Yimen counties from western Yunnan, and Yuxi city from central Yunnan.

After clone correction, limited but unequivocal evidence of recombination at nine STR loci was found in total *A. fumigatus* samples and in individual subpopulations, except for the SC population (PrC = 1, *P* = 0.056; rBarD = 0.297, *P* < 0.001) (Table S2).

Analysis of molecular variance (AMOVA) based on different isolation sources and geographical regions revealed that most of the genetic variation (95%) existed within local populations (*P* = 0.001), while only 1% of the genetic variation was due to differences among different local populations (*P* = 0.002) (Table 4). However, when comparing clinical samples with environmental samples from various habitats, such as farmland, forests, and cultivation areas, we found a higher level of gene flow among geographical populations (Nm = 18.721) than between clinical and environmental populations (Nm = 9.538). Furthermore, the categorization of samples according to clinical and environmental sources contributed more significantly to the total genetic variation (4%) compared to the division based on different geographical regions (1%) (Table 4).

**TABLE 4:** Summary results of AMOVA among regions of the *A. fumigatus* isolates from different sources

Source	df	SS	MS	Est. Var.	%	PhiRT	*P*	Nm
Clinic-environment								
Among regions	1	30.186	30.186	0.183	4%	0.044	0.001	9.538
Among pops	6	28.868	4.811	0.023	1%	0.006	0.002	
Within pops	337	1325.946	3.935	3.935	95%	0.050	0.001	
Total	344	1385.000		4.141	100%			
Different areas								
Among regions	3	24.113	8.038	0.000	0%	−0.020	1.000	18.721
Among pops	4	34.942	8.735	0.186	5%	0.045	0.001	
Within pops	337	1325.946	3.935	3.935	95%	0.026	0.001	

Further examination of the degree of genetic differentiation among the populations revealed significant genetic differences in 20 of the 28 pairs. WC and WE exhibited the most significant genetic differentiation (PhiPT = 0.071, *P* = 0.001), while CE and WE showed the least differentiation (PhiPT = 0.011, *P* = 0.007) (Table S3). Notably, significant genetic differentiation was observed between the WE population and the other seven populations (*P* < 0.05).

**Fig 1:**
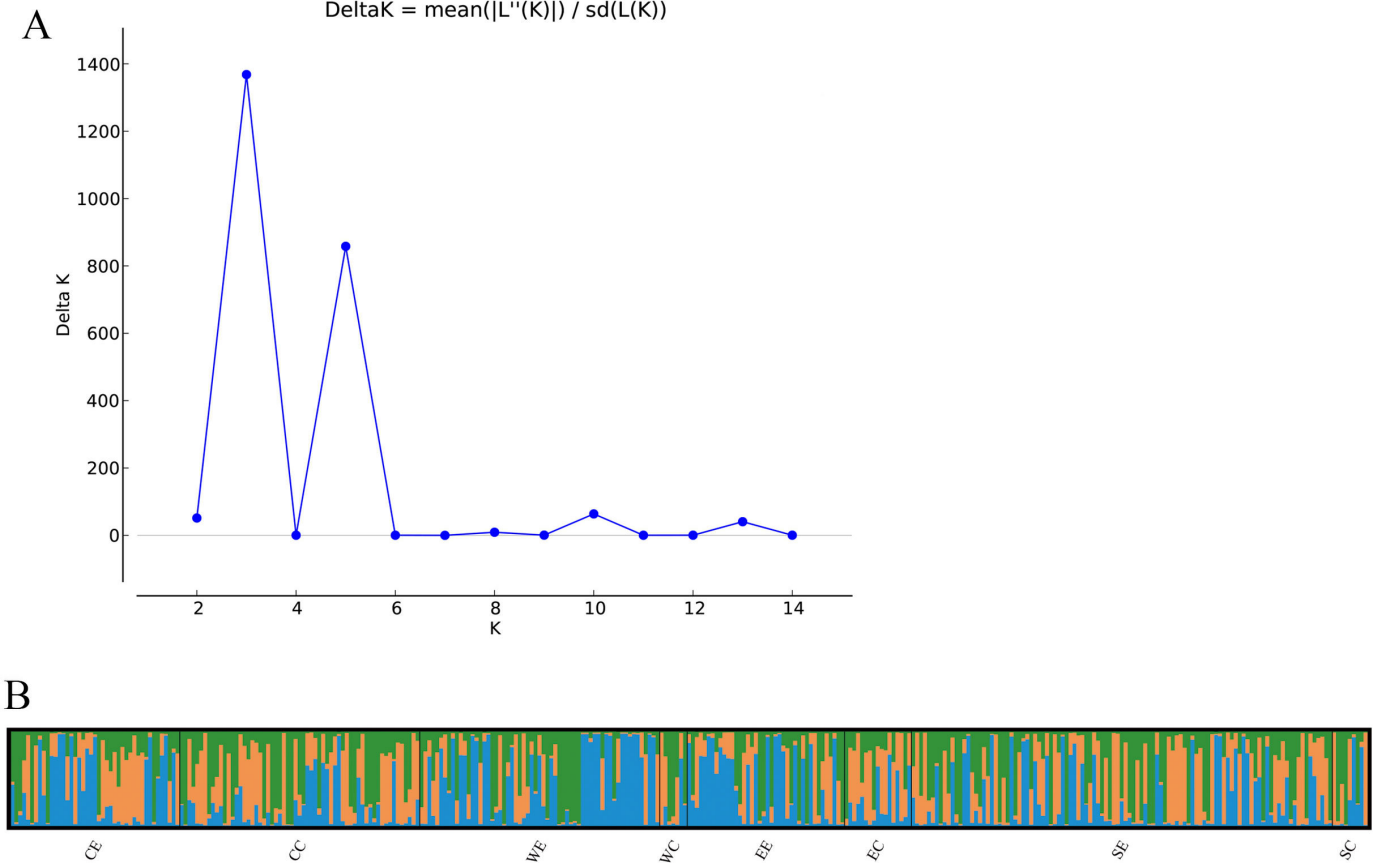
Genetic structuring results obtained from the STRUCTURE analysis. Plot of *K* against delta *K* (**A**) and analyses (**B**) for eight populations. The program STRUCTURE version 2.3.5 was used to determine the optimal number (*K*) of genetic clusters, and the colors represent different genetic clusters.

To identify potentially distinct genetic groups in *A. fumigatus* samples, we analyzed the number of possible genetic clusters using STRUCTURE software. The analysis inferred three clusters (*K* = 3), as indicated by the lowest standard deviation of posterior probability for that *K* value (Fig. 1A). These three genetic clusters were broadly distributed among both environmental and clinical strains and across different geographic populations. For example, the environmental populations WE and EE displayed slightly varied proportions of different genetic elements (Fig. 1B).

**Fig 2:**
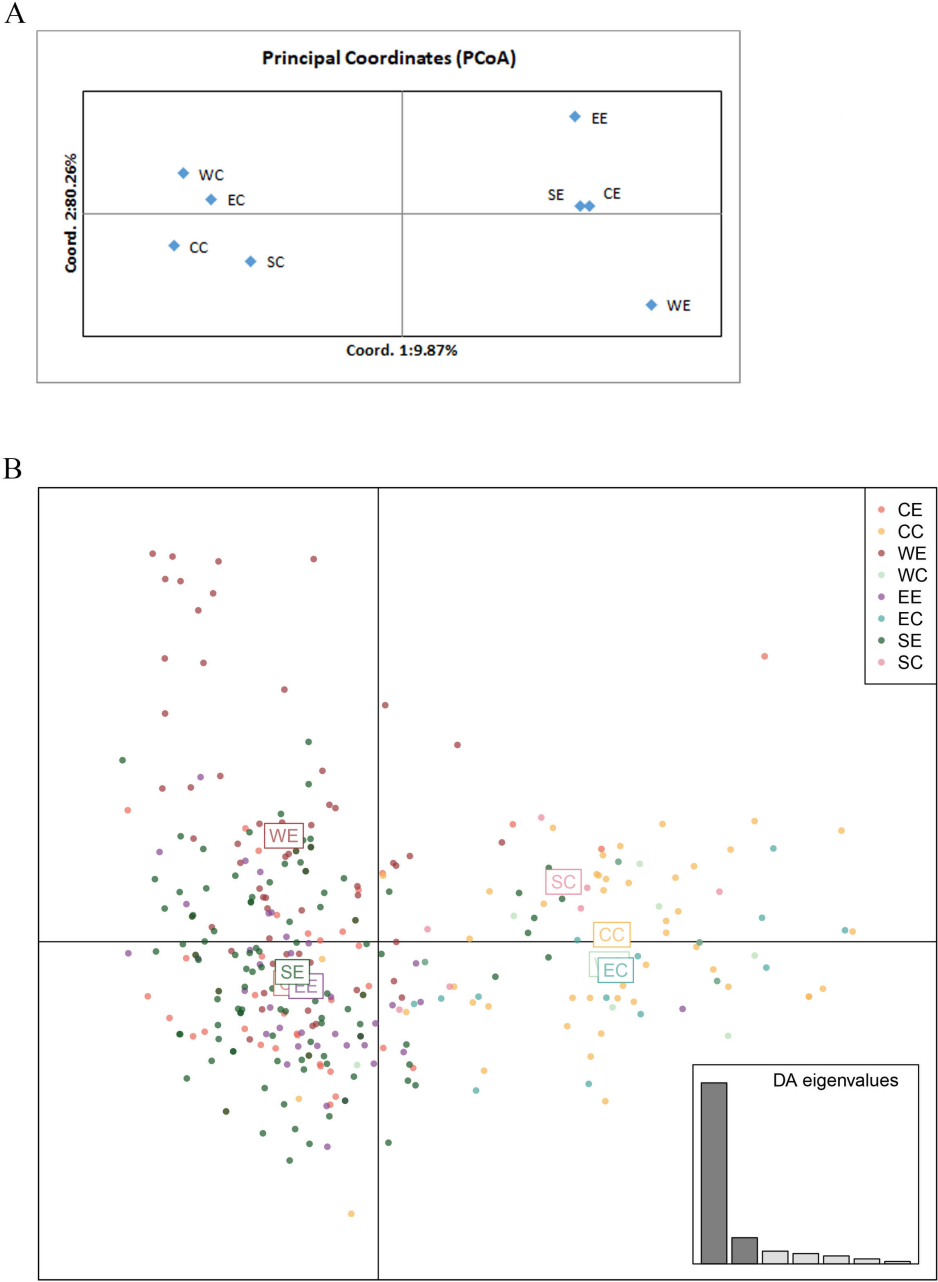
Results of PCoA based on pairwise population genetic distances (**A**) and DAPC analysis between eight populations (**B**).

Principal coordinate analysis, using average population haplotype genetic distances, showed that the first two coordinates together explained 90.13% of the total variation (PC1 = 80.26%, PC2 = 9.87%) (Fig. 2A). Although genetic variation differentiation was more significant between clinical and environmental isolates than among geographical distributions, strains from the southern area (SC and SE) clustered more closely than those from the western area (WC and WE), as evident in the results of principal component discriminant analysis (Fig. 2B) and genetic differentiation between population pairs. A few clinical samples were grouped into the clusters of environmental samples, although most were independently distributed in the PCoA and DAPC analyses (Fig. 2).

**Fig 3:**
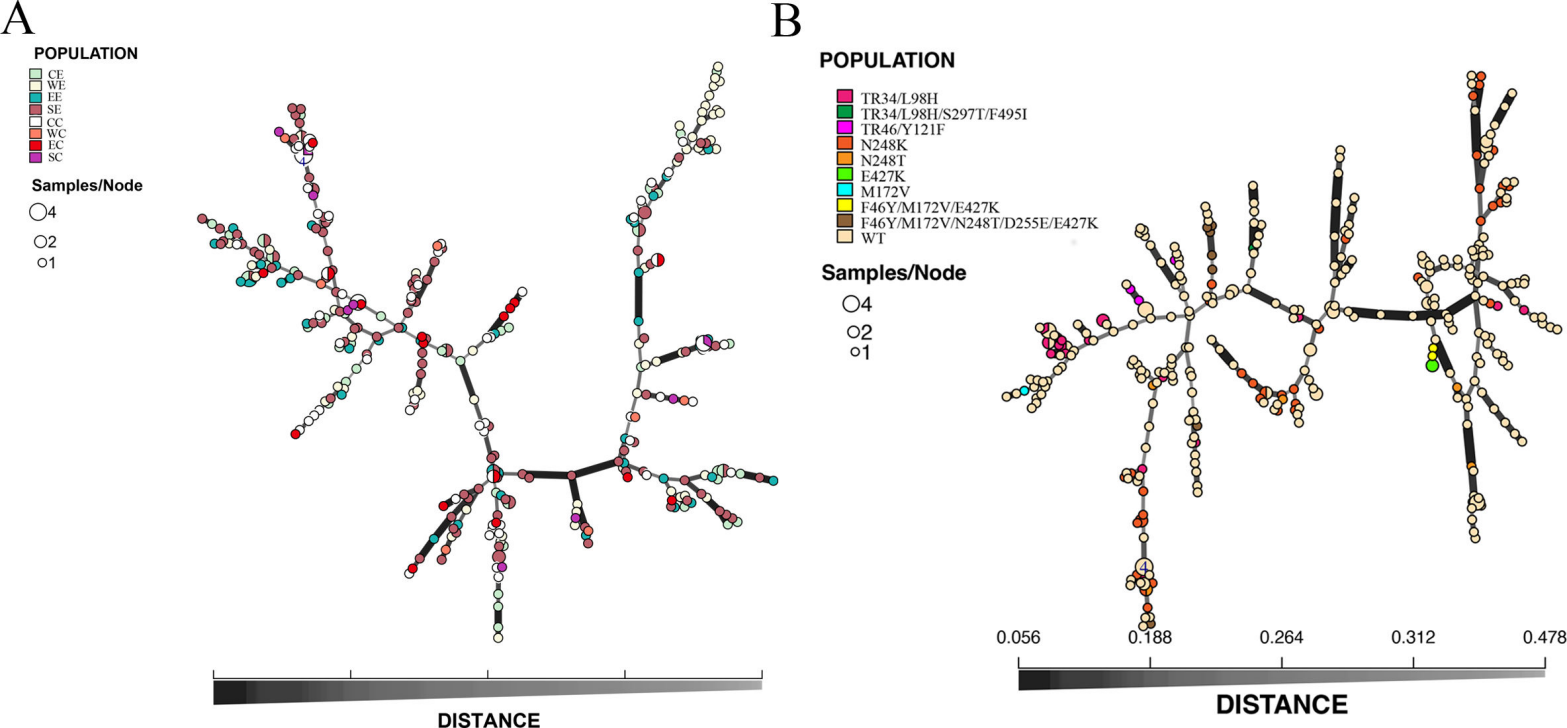
Minimum spanning tree showing genotype relationships among different geographic populations in Yunnan Province (**A**) and the origin of mutation types in the *cyp51A* gene (**B**). Each circle corresponds to a unique genotype, and the size of the circle proportionally represents the number of isolates with that genotype. Connecting lines correspond to the number of differences between genotypes.

Clustering analysis using the unweighted pair group method with arithmetic mean revealed that some clinical *A. fumigatus* isolates clustered into the same subclades as environmental isolates (Fig. 3).

**Fig 4:**
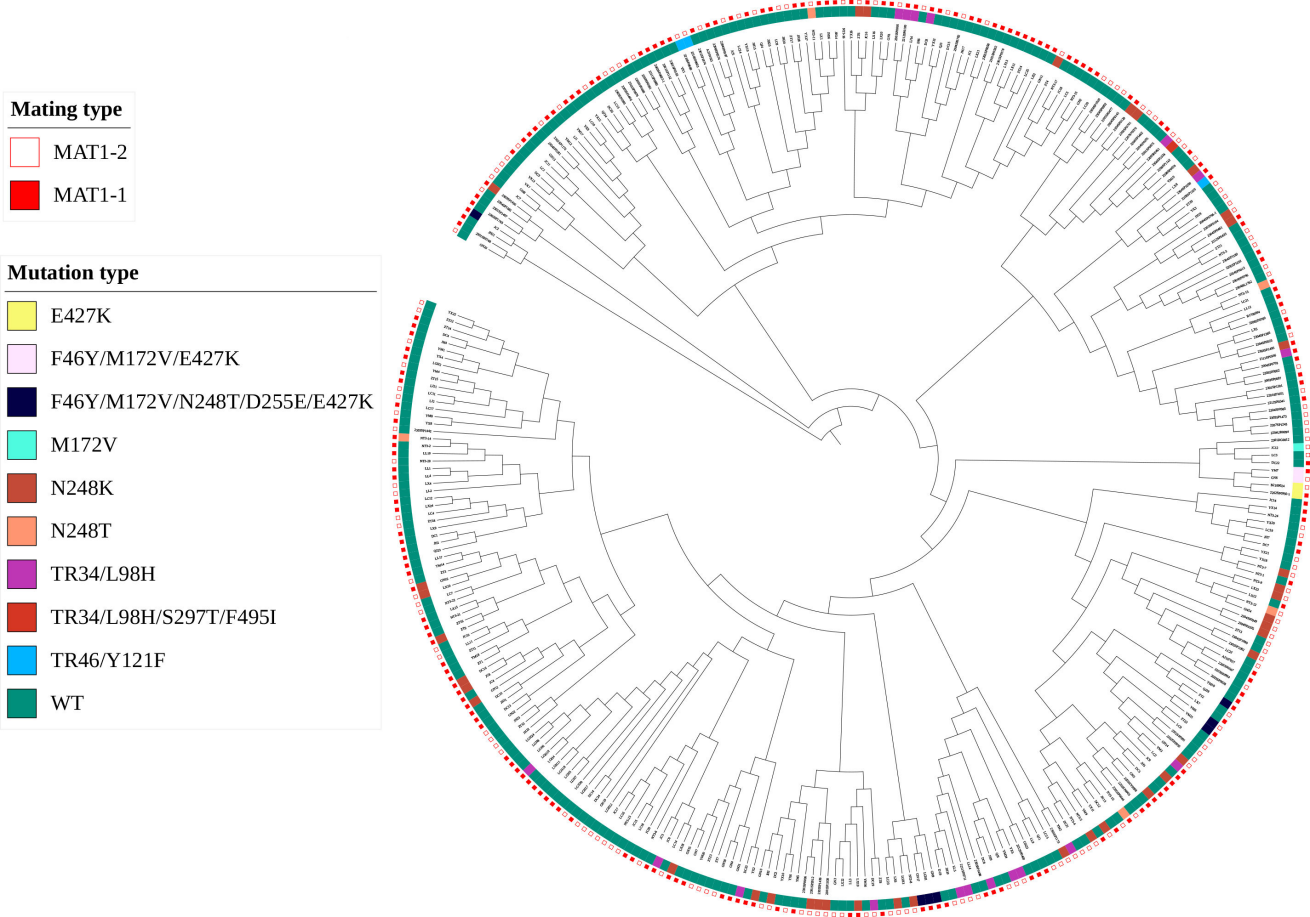
Phylogenetic tree of *A. fumigatus* strains under study. Maximum likelihood phylogenetic circular phylograms based on STR data were constructed using UDPG and Interactive Tree of Life programs. Orange branches in the figure represent environmental *A. fumigatus* isolates, and black represents clinical *A. fumigatus* isolates.

To better visualize the relationships among the eight populations and the origins of the azole resistance-associated mutations in the *cyp51A* gene, we described the genotypic relationships of 345 *A*. *fumigatus* strains based on Bruvo distances between STR genotypes by constructing an MSN tree. MLGs of different origins (clinical and environmental, or different geographical areas) as well as triazole resistance-associated *cyp51A* gene mutations were then superimposed on the MSN tree. The results revealed that most genotypes from different geographic regions and origins (environmental or clinical) are widespread and mixed on the MSN tree (Fig. 4A). Moreover, except for N248K and TR_34_/L98H, which had a large number of candidates (38 and 18, respectively), other mutation types had only a limited distribution, and they are often distributed at the end of the branches despite their geographic regions and origins (Fig. 4B).

### Distribution of mating types

The relationship between environmental and clinical strains, cyp51A mutations, and mating types was analyzed. Among 345 strains, MAT1-1 and MAT1-2 were nearly equally distributed (158 MAT1-1 and 187 MAT1-2). Clinical isolates had 53.19% MAT1-1 and 46.86% MAT1-2, while environmental isolates had 43.03% MAT1-1 and 56.97% MAT1-2, with no significant differences (*P* = 0.1846 and *P* = 0.0781). The mating type ratio was also close to 1:1 in both mutated (119:149) and wild-type strains (39:38).

In resistant strains, all three TR46/Y121F mutants had two MAT1-1 and one MAT1-2. The MAT1-1 to MAT1-2 ratio remained close to 1:1 in strains with TR34/L98H and TR34/L98H/S297T/F495I mutations. Overall, the mating type ratio in strains with tandem repeat mutations in the cyp51A promoter was also about 1:1.

## DISCUSSION

In this study, clinical isolates of *A. fumigatus* were collected from patients living in various regions across Yunnan Province, southwestern China; the genetic diversity and differentiation of the species between clinical and environmental samples, as well as the antifungal drug sensitivity patterns and resistance mechanisms were compared, in order to unveil environmental transmission risks in these areas with high prevalence of azole resistance.

### Differences in diversity and unique genetic elements

Clinical *A. fumigatus* isolates showed slightly higher diversity (uh = 0.887) than environmental isolates (uh = 0.865), but environmental isolates exhibited more unique genetic elements, possibly influenced by sample size and environmental conditions. Geographic and climatic factors shape the genetic structure of environmental populations, likely contributing to the accumulation of locally adapted mutations and genotypes within and among local environmental populations (45).

The differences in diversity and unique genetic elements observed between clinical and environmental isolates can be attributed to several factors. First, the human body may impose selective pressures on *A. fumigatus*, adapting to patient conditions, while the environment provides more favorable growth conditions. Second, while clinical populations in this study were categorized based on patients’ residences, it remains uncertain whether there has been long-distance environmental-to-human or human-to-human transmission of the infection. Factors such as long-distance travel, interpersonal interactions, and the trade of non-local food and agricultural products may contribute to the transmission dynamics, which increases the complexity of clinical genotypes rather than the environmentally residential ones. Even if *A. fumigatus* isolates infecting patients originate from nearby environmental sources, the increased mobility of individuals and the broader range of potential transmission routes may lead to more complex genetic exchanges among clinical isolates. Third, environmental isolates from isolated agricultural environments may experience limited genetic exchange, leading to simpler genetic structures.

Studies have reported variability in genetic composition across different *A. fumigatus* origins (58), with clinical isolates showing reactivation of virulence traits (59) and potential genotype-environment interactions, leading to diversity differences and impacts on drug resistance (60). In our study, we observed significant differences in drug resistance frequencies between clinical and environmental isolates. This genetic variation between environmental and clinical groups underscores the potential impact on the frequency of isolate resistance to first-line therapeutic drugs.

Then, our findings regarding unique genetic elements differ from previous reports on Kunming greenhouses (37), where a higher proportion of unique genetic elements was observed, likely due to the isolated nature of greenhouse environments, which limit gene flow and encourage the development of distinct genotypes. Kunming greenhouses, situated in central Yunnan province, exhibit distinct features that promote the development of unique genetic traits in *A. fumigatus* populations. Previous research has highlighted greenhouses as significant contributors to the genetic diversity of fungal populations, including *A. fumigatus* and *Alternaria alternata*, even among greenhouses located in close proximity to each other (37, 61). Unlike open farmland, greenhouses are characterized by intensive cultivation practices, confined spaces, higher temperatures, and humidity levels. Each greenhouse functions as an isolated microenvironment, creating barriers to gene flow and exchange among fungal populations, thus may also act as reservoirs for drug-resistant strains, which could be transmitted to humans. In our unpublished study, we observed similarities between the genotypes of drug-resistant *A. fumigatus* strains isolated from greenhouse soils and those obtained from nearby clinical patients. This underscores the importance of assessing the diversity and spread of drug-resistant genotypes originating from greenhouses.

Finally, we compared our Yunnan clinical samples to those from other parts of China. The frequency of unique genotypes among clinical *A. fumigatus* isolates in Yunnan was higher than reported in Shanghai (72.41%) (62) and the eastern, central, and western regions of China in 2017 (42.86%) (63). Though small sample set (13) was isolated in 2018 (64), 83.37% of unique genotype found in Shanghai and Jiangxi Province in 2023 were lower than our data in Yunnan (65).

### Genetic differentiation and widespread allele sharing between the clinical and environmental populations

Our study revealed significant differentiation between clinical and environmental populations, surpassing the contribution of geographical origins of isolates. This suggests that recent transmission chains have not formed within the same geographic areas. As discussed in the earlier published paper, while it has been shown that *A. fumigatus* spores in different geographic groups can be transmitted by air and water currents (52), the unique alpine geography and varying degrees of human interaction in agricultural and natural environments contribute to this differentiation, and we found no clear geographical factors influencing genetic variation in clinical settings. Barber et al. observed a significant enrichment of genotypes among clinical isolates compared to environmental *A. fumigatus* isolates, suggesting that this genetic background might be better suited for survival and proliferation in the human environment and during infection (44). Phylogenetic analyses revealed independent evolutionary signals in both clinical and environmental strains, with clinical isolates demonstrating broad-spectrum fitness. Future research should focus on acquiring more local clinical strains to further explore interactions between *A. fumigatus* and host environments.

Our investigation found no correlation between cyp51A gene mutations and mating type genes, indicating that MAT genes do not influence drug resistance emergence. Limited recombination was detected across nine STR loci, but an equal distribution of mating genes (MAT1-1 and MAT1-2) suggests potential for sexual reproduction in both populations. Given the observed allele numbers and assuming recombination, the total possible number of multilocus genotypes at the nine microsatellite loci in both of the environmental and clinical population of *A. fumigatus* from Yunnan is 17 × 25 × 28 ×49 × 32 × 38 × 20 × 13 × 15 =2,765,293,440,000. This potential for recombination led to rapid shuffling and recombination of the nine STR loci we tested, and it was difficult to find allele similarities due to the environmental and clinical time lag in sample collection and isolation. The extremely high number of possible genotypes leads to the extremely low probability of having strains with identical genotypes at the nine loci by chance and not due to clonal reproduction. However, we still found the sharing of two to six alleles in clinical and environmental strains, further suggesting the existence of environmental and clinical transmission chains.

Notably, 83.12% of STR alleles (197/237) were shared between clinical and environmental populations. Allele #4 at locus 4C, previously rare globally, was prevalent in our study, as were alleles #19 at locus 2A and #11 at locus 3B, which had higher frequencies in environmental strains. These evidences suggest potential transmission of this allele from the environment to humans in Yunnan.

Furthermore, we observed partial genotype similarities in pairs of clinical and environmental isolates, particularly in Guangnan County, located in the southeast of Yunnan, at the junction of Yunnan, Guangxi, and Guizhou provinces. Notably, pairs 2211SP0711 (Shizong County from Qujing city) and LL5 (Luling County from Qujing City) exhibited identical alleles and resistance profiles, and the environmental sampling site is only 71 km away from the resident of the patient, highlighting the proximity of environmental samples to clinical cases. This raises concerns about the potential for local transmission of drug-resistant strains.

Comparative analyses revealed 42.42% genotype sharing among *A. fumigatus* isolates in Iran and similar findings in France, indicating widespread genetic distribution (2, 12). Sewell et al. frequently noted that azole-resistant *A. fumigatus* clones could share the exact same STRAf genotype from both environmental and clinical locations (42). Fisher et al. highlighted that clinical isolates originated from a broader environmental population, with patients infected with resistant isolates transmitted from the environment (66). Despite resistance rate differences, some similarly genotyped isolates exhibited similar minimum inhibitory concentrations to azole treatments, suggesting a link between environmental use of azole fungicides and the emergence of resistant clinical isolates. Notably, the isolation of azole-resistant *A. fumigatus* does not significantly diminish its clinical relevance (66). However, the selection pressure on environmental strains of *A. fumigatus* may significantly impact the clinical practice of antifungal drug therapy (67).

Meanwhile, the frequent sharing of alleles but limited or no overlap in MLGs indicated frequent recombination in nature. The analysis of molecular variance indicated reduced gene flow between clinical and environmental samples (Nm = 9.538), while evidence of non-random recombination and potential for sexual reproduction were observed in the equal MAT ratios. Previous studies have indicated that recombination could accelerate adaptation to novel environmental conditions and the distribution of drug-resistant alleles, including adaptation from the environment to human infection (40, 68, 69). In the future, surveillance and early warning systems for the spread of environmental resistant genotypes need to be established and strengthened in Yunnan.

### Antifungal drug sensitivity profiles

Antifungal drug sensitivity patterns are crucial for informing treatment strategies. Antifungal agents are typically classified into various groups, including polyenes, fluoropyrimidine analogs, azoles, echinocandins, and others (67). In our study, we found that 71.28% of clinical *A. fumigatus* isolates were resistant to POS, 15.96% and 8.51% isolates have MIC values ≥2 and 4 mg/L resistant to ITR, respectively, which were notably higher than the 2.5% (8/317) ITR resistance reported by Chen et al. (16) across 12 provinces in China, as well as the 4.4% (7/159) ITR resistance reported in central and southeastern China by Tavakoli et al. (60). Additionally, between January 2019 and April 2021, a high susceptibility to POS (100%), ITR (100%), and VOR (95.89%) was just observed among clinical *A. fumigatus* isolates in Anhui Province, central China (16, 70, 71), which was also lower than ITR and VOR resistance in our findings.

Resistance patterns to ITR and VOR varied regionally; the highest frequencies were in the eastern clinical group, while environmental resistance often exceeded that of clinical strains. Notably, no strains resistant to ITR, VOR, CAS, MIF, or AMB were detected in clinical samples from western regions.

Frequent use of triazole fungicides in agricultural environments can lead to the emergence of AR*Af* (72). Importantly, among the clinical samples, we identified multi-drug resistance patterns, including but not limited to cross-resistance to ITR and VOR, with varying resistance patterns based on MIC levels. Furthermore, the incidence of azole multidrug resistance in clinical isolates in our study was higher than that reported by Yang et al. in 2021 for clinical isolates from the First Hospital of Peking University in China (2.70%, 12/445) (73), as well as in 2022 from a hospital in Anhui, China (4.11%) (71), and in 2023 from Zhongshan Hospital of Fudan University in Shanghai, China (4/80) (35). We hypothesize that extensive genetic exchange between local environmental and clinical isolates in Yunnan has heightened azole resistance prevalence.

Additionally, the frequency of AMB resistance in our study was 7.45% (7/94). In contrast, a review of studies from 2010 to 2020 indicated only 2% resistance among 17,494 strains globally (74). The highest AMB resistance reported during the decade was 12.07% (7/58) in Korea (75) (76) and 29.55% (26/88) in a clinical setting in Anhui, China (71). Given that the mortality rate of invasive aspergillosis treated with AMB ranges from 65% to 71% (77, 78), these findings highlight the urgent need for careful drug selection and treatment strategies in managing aspergillosis in Yunnan Province.

### Prevalence and specificity of *cyp51A* mutations

The prevalence and specificity of *cyp51A* mutations and their correlation with MICs provided insights into antifungal resistance mechanisms, the impact of geographic distribution, and implications for treatment strategies. In our study, we found N248K mutation was widespread in both clinical and environmental samples but showed fewer significant associations with specific MIC values. N248T, found only in environmental samples, was significantly associated with lower MIC values for ITR and VOR. Among 345 samples, ~6% exhibited tandem repeat insertions in the promoter region of the *cyp51A* gene, including TR_34_ (5.22%) and TR_46_ (0.87%). The TR_34_/L98H mutation was more prevalent in environmental samples (12) compared to clinical ones (6), while TR_46_ mutations were found exclusively in clinical samples. We further observed that the TR_34_/L98H mutation was significantly associated with ITR and VOR resistance in both clinical and environmental samples, though the MIC value ranges differed. TR_34_/L98H was identified as the primary resistance mechanism of AR*Af* in Yunnan by environmental isolating (37, 45). This distribution is consistent with reports from other regions of China and globally (18, 42, 79, 80), suggesting potential transmission routes from environmental to clinical settings. While clinical *A. fumigatus* isolates with the TR_46_/121F mutation have been reported in China since 2016 (16), and the mutation has only been detected in small numbers in subsequent environmental studies (37, 40, 45), was significantly correlated with high MIC values (>8 mg/L) against VOR, indicating risks of environmental acquisition of drug-resistant strains.

We found significant correlations between observed mutations and isolate frequencies across different MIC ranges, particularly with tandem repeat mutations. The TR34/L98H/S297T/F495I combination was associated with higher MIC values for ITR and VOR in clinical samples, while the F46Y/M172V/E427K combination was linked to higher MIC values for ITR in environmental samples. Geographical preferences for mutations were observed, with TR34 prevalent in central and western Yunnan, and TR46 predominantly found in central Yunnan.

Nine non-synonymous substitutions were identified, with Y121F, S297T, and F495I exclusive to clinical samples, while F46Y, M172V, and D255E were found only in environmental samples. Certain mutation combinations (e.g., F46Y/M172V/N248T/D255E/E427K) showed significant associations with antifungal resistance in environmental samples. The TR34/L98H mutation was significantly associated with low MIC values for several clinical drugs (e.g., ANI, POS), while N248K mutations were associated with resistance to ANI, MIF, and AMB specifically in clinical strains.

Interestingly, the ratio of mating types MAT1-1 and MAT1-2 in tandem repeat mutations was close to 1:1, consistent with studies in the US, France, and the UK (44, 81–83). However, the distribution of mating types differed significantly from that of tandem repeat mutations (*P* = 0.0244, *P* = 0.0094), with a higher proportion of MAT1-2 in environmental and susceptible strains.

Indeed, our MSN analysis showed a wide distribution of clinical and environmental STR genotypes, azole-resistant and susceptible strains, and various mutation types, suggesting that the emergence of highly resistant phenotypes and specific mutations in *A. fumigatus* from Yunnan is a multi-origin process influenced by the province’s unique geography and natural recombination. Some mutations may provide adaptive advantages despite not being significantly correlated with high MIC values. Furthermore, strains with elevated MIC values or cross-resistance to multiple drugs may still be wild type for cyp51A, warranting further investigation into their resistance mechanisms.

### Conclusion

Overall, the frequency of azole resistance among clinical *A. fumigatus* isolates in Yunnan was lower than that observed in environmental samples but higher compared to other regions of China. While we attribute the genetic differentiation between clinical and environmental isolates primarily to the unique geomorphology of Yunnan, extensive human mobility and trade have also facilitated genetic exchange among *A. fumigatus* populations. The substantial exchange of alleles has contributed to the selection of azole-resistant clinical *A. fumigatus* isolates, with the high number of shared alleles suggesting a common environmental origin for these patients. Given the public health threat posed by fungal pathogens and the limited availability of antifungal drugs for treating invasive infections, it is imperative to break the environmental-clinical chain of *A. fumigatus* transmission. This can be achieved by placing immunocompromised patients in protected environments to minimize exposures and prevent secondary infections. Additionally, reducing exposure to environments with high risk of infection, such as gardening, composting, and farm markets, is essential (84).

Furthermore, TR_34_ and TR_46_ mutations have been linked to environmental fungicide exposure, with studies confirming that resistant *A. fumigatus* strains originating in the environment can cause drug-resistant infections in humans (85). Hence, urgent measures are needed to control the use of antifungal drugs in clinical settings and azole fungicides in agriculture. Finally, research efforts should focus on defining the epidemiology and management of AR*Af* and enhancing surveillance of fungal pathogens. This is particularly crucial as new patient populations recognize the increased risk of invasive aspergillosis (86).

## SUPPLEMENTAL MATERIAL

Table S1 to S3 spectrum.01594-24-s0001.docx Table S1: Genetic diversity of different local populations. Table S2: Multilocus linkage disequilibrium analyses of eight *A. fumigatus*. Table S3: Pairwise genetic differentiations between eight populations of *A. fumigatus*.

Table S4 spectrum.01594-24-s0002.xlsx Detailed information of strains.

ASM does not own the copyrights to Supplemental Material that may be linked to, or accessed through, an article. The authors have granted ASM a non-exclusive, world-wide license to publish the Supplemental Material files. Please contact the corresponding author directly for reuse.
