# Supplementary material for: Unveiling environmental transmission risks: comparative analysis of azole resistance in Aspergillus fumigatus clinical and environmental isolates from Yunnan, China
Source: Microbiol Spectr. 2024 Oct 29;12(12):e01594-24. doi: 10.1128/spectrum.01594-24 (PMC11619395; doi:10.1128/spectrum.01594-24)
Supplement: Table S1 to S3 — Table S1: Genetic diversity of different local populations. Table S2: Multilocus linkage disequilibrium analyses of eight A. fumigatus. Table S3: Pairwise genetic differentiations between eight populations of A. fumigatus. [file spectrum.01594-24-s0001.docx]

**Table S1 Genetic diversity of different local populations**

| Pop |  | N | Na | Ne | I | h | uh |
| --- | --- | --- | --- | --- | --- | --- | --- |
| CE | Mean | 42.333 | 15 | 9.7 | 2.362 | 0.868 | 0.889 |
|  | SE | 0.553 | 1.986 | 1.539 | 0.168 | 0.027 | 0.027 |
|  |  |  |  |  |  |  |  |
| CC | Mean | 59.889 | 16.111 | 10.125 | 2.371 | 0.867 | 0.882 |
|  | SE | 0.655 | 2.189 | 1.859 | 0.188 | 0.026 | 0.027 |
|  |  |  |  |  |  |  |  |
| WE | Mean | 58.889 | 16.111 | 9.179 | 2.279 | 0.83 | 0.844 |
|  | SE | 1.306 | 2.098 | 2.196 | 0.205 | 0.044 | 0.044 |
|  |  |  |  |  |  |  |  |
| WC | Mean | 7 | 5.444 | 5.062 | 1.573 | 0.753 | 0.878 |
|  | SE | 0 | 0.58 | 0.702 | 0.149 | 0.048 | 0.056 |
|  |  |  |  |  |  |  |  |
| EE | Mean | 39.889 | 13.889 | 9.093 | 2.241 | 0.832 | 0.854 |
|  | SE | 0.111 | 1.975 | 1.801 | 0.206 | 0.042 | 0.043 |
|  |  |  |  |  |  |  |  |
| EC | Mean | 16.667 | 9.444 | 7.162 | 2.053 | 0.84 | 0.893 |
|  | SE | 0.236 | 0.801 | 0.855 | 0.115 | 0.023 | 0.024 |
|  |  |  |  |  |  |  |  |
| SE | Mean | 106.333 | 20.222 | 10.78 | 2.459 | 0.863 | 0.871 |
|  | SE | 0.373 | 3.471 | 2.132 | 0.218 | 0.033 | 0.033 |
|  |  |  |  |  |  |  |  |
| SC | Mean | 8.556 | 5.778 | 5.088 | 1.654 | 0.789 | 0.893 |
|  | SE | 0.176 | 0.521 | 0.437 | 0.1 | 0.022 | 0.024 |
|  |  |  |  |  |  |  |  |
| Total | Mean | 42.444 | 12.75 | 8.274 | 2.124 | 0.83 | 0.876 |
|  | SE | 3.695 | 0.881 | 0.584 | 0.069 | 0.012 | 0.012 |

**Table S2** **Multilocus linkage disequilibrium analyses of eight *A. fumigatus***

| Pop |  | PrCompat |  | rBarD |
| --- | --- | --- | --- | --- |
| All(n=318) |  | 0(*p*=1) |  | -0.148(p<0.001) |
| CC(n=50) |  | 0(*p*=1) |  | 0.039(*p*=＜0.001) |
| CE(n=34) |  | 0.056(*p=*0.359) |  | 0.063(*p*<0.001) |
| EC(n=17) |  | 0.668(*p*=0.382) |  | 0.062(*p*<0.001) |
| EE(n=38) |  | 0.056(*p*=0.285) |  | 0.046(*p*＜0.001) |
| SC(n=9) |  | 1.000(*p*=0.056) |  | 0.297(*p＜*0.001) |
| SE(n=105) |  | 0(*p*=1) |  | 0.025(*p*<0.001) |
| WC(n=7) |  | 0.972(p=0.922) |  | -0.066(0.898) |
| WE(n=58) |  | 0(*p=1*) |  | 0.081(*p*<0.001) |

**Table S3** **Pairwise genetic differentiations between eight populations of *A. fumigatus***

| CE | WE | EE | SE | CC | WC | EC | SC |  |
| --- | --- | --- | --- | --- | --- | --- | --- | --- |
|  | 0.003 | 0.175 | 0.455 | 0.001 | 0.008 | 0.001 | 0.013 | CE |
| 0.011 |  | 0.001 | 0.001 | 0.001 | 0.001 | 0.001 | 0.005 | WE |
| 0.004 | 0.018 |  | 0.010 | 0.001 | 0.011 | 0.001 | 0.002 | EE |
| 0.000 | 0.013 | 0.009 |  | 0.001 | 0.005 | 0.001 | 0.004 | SE |
| 0.042 | 0.062 | 0.048 | 0.045 |  | 0.449 | 0.478 | 0.453 | CC |
| 0.046 | 0.071 | 0.040 | 0.045 | 0.000 |  | 0.463 | 0.477 | WC |
| 0.044 | 0.062 | 0.047 | 0.044 | 0.000 | 0.000 |  | 0.232 | EC |
| 0.028 | 0.048 | 0.041 | 0.030 | 0.000 | 0.000 | 0.010 |  | SC |
